# The Contribution of AMPA and NMDA Receptors to Persistent Firing in the Dorsolateral Prefrontal Cortex in Working Memory

**DOI:** 10.1523/JNEUROSCI.2121-19.2020

**Published:** 2020-03-18

**Authors:** Bram van Vugt, Timo van Kerkoerle, Devavrat Vartak, Pieter R. Roelfsema

**Affiliations:** ^1^Department of Vision & Cognition, Netherlands Institute for Neuroscience, 1105 BA, Amsterdam, The Netherlands,; ^2^Cognitive Neuroimaging Unit, Commissariat à l'Énergie Atomique et aux Énergies Alternatives, Direction des Sciences du Vivant/Institut d'Imagerie Biomédicale, Institut National de la Santé et de la Recherche Médicale, NeuroSpin Center, Université Paris-Sud, Université Paris-Saclay, 91191 Gif-sur-Yvette, France,; ^3^Department of Integrative Neurophysiology, Center for Neurogenomics and Cognitive Research, Vrije Universiteit, 1081 HV Amsterdam, The Netherlands, and; ^4^Psychiatry Department, Academic Medical Center, 1105 AZ Amsterdam, The Netherlands

**Keywords:** AMPAR, macaque monkey, NMDAR, PFC, working memory

## Abstract

Many tasks demand that information is kept online for a few seconds before it is used to guide behavior. The information is kept in working memory as the persistent firing of neurons encoding the memorized information. The neural mechanisms responsible for persistent activity are not yet well understood. Theories attribute an important role to ionotropic glutamate receptors, and it has been suggested that NMDARs are particularly important for persistent firing because they exhibit long time constants. Ionotropic AMPARs have shorter time constants and have been suggested to play a smaller role in working memory. Here we compared the contribution of AMPARs and NMDARs to persistent firing in the dlPFC of male macaque monkeys performing a delayed saccade to a memorized spatial location. We used iontophoresis to eject small amounts of glutamate receptor antagonists, aiming to perturb, but not abolish, neuronal activity. We found that both AMPARs and NMDARs contributed to persistent activity. Blockers of the NMDARs decreased persistent firing associated with the memory of the neuron's preferred spatial location but had comparatively little effect on the representation of the antipreferred location. They therefore decreased the information conveyed by persistent firing about the memorized location. In contrast, AMPAR blockers decreased activity elicited by the memory of both the preferred and antipreferred location, with a smaller effect on the information conveyed by persistent activity. Our results provide new insights into the contribution of AMPARs and NMDARs to persistent activity during working memory tasks.

**SIGNIFICANCE STATEMENT** Working memory enables us to hold on to information that is no longer available to the senses. It relies on the persistent activity of neurons that code for the memorized information, but the detailed mechanisms are not yet well understood. Here we investigated the role of NMDARs and AMPARs in working memory using iontophoresis of antagonists in the PFC of monkeys remembering the location of a visual stimulus for an eye movement response. AMPARs and NMDARs both contributed to persistent activity. NMDAR blockers mostly decreased persistent firing associated with the memory of the neuron's preferred spatial location, whereas AMPAR blockers caused a more general suppression. These results provide new insight into the contribution of AMPARs and NMDARs to working memory.

## Introduction

Working memory refers to the ability to store and manipulate information over short periods of time, on the order of seconds (Baddeley, 2012). In many situations, we have to briefly remember what we perceived, and we then store this information in working memory; whereas in other situations, working memories are retrieved from long-term memory. The ability to store and manipulate information is crucial for cognition in daily life (Christophel et al., 2017), and a deeper understanding of its neural basis would be of great medical and social significance because disorders, such as schizophrenia (Driesen et al., 2008) and Alzheimer's disease (Schroeter et al., 2012), degrade the quality of working memory (Riley and Constantinidis, 2015).

**Figure 1.**
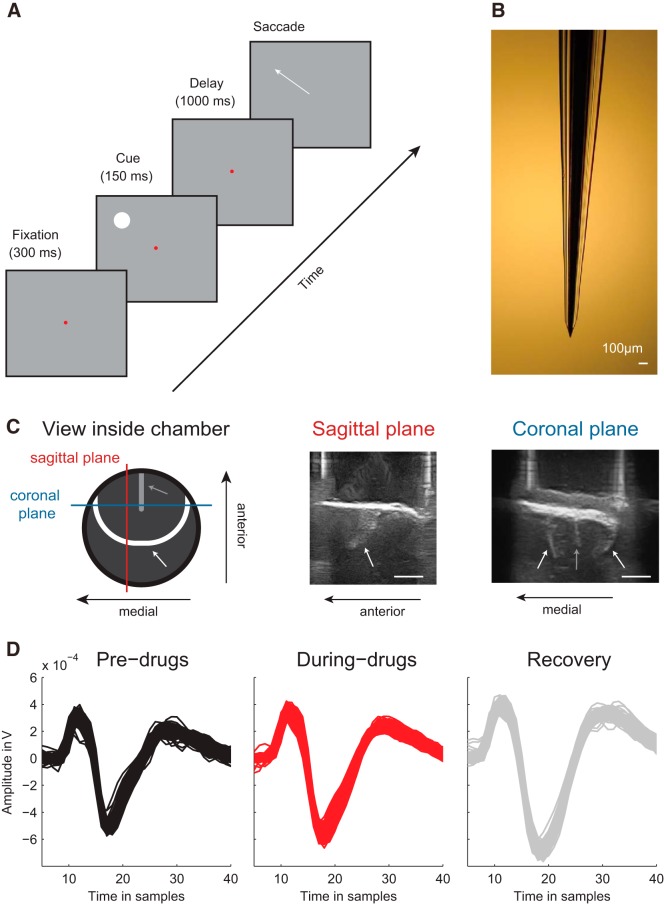
Microiontophoresis in dlPFC during an oculomotor delayed response task. ***A***, After a 300 ms fixation epoch, a white spatial cue was presented for 150 ms, and the monkey maintained fixation for another 1000 ms. After the fixation point disappeared, the monkey made a saccade to the memorized location. ***B***, Three barrel glass electrode used for microiontophoresis. The central barrel contained a tungsten electrode, and the side barrels were used for the iontophoretic application of drugs. ***C***, Left, Schematic of the inside of the chamber of Monkey B, with the estimated location of the arcuate sulcus (white thick line) and principal sulcus (gray thick line). Colored lines indicate the location of ultrasound images. Middle, Sagittal ultrasound slices of this recording chamber. Right, Coronal ultrasound slices of this recording chamber. White arrows point to the arcuate sulcus. Gray arrows point to the principal sulcus. Scale bars, 5 mm. ***D***, Example wave shape of a well-isolated single unit during the block of trials before drug delivery (black), during drug delivery (red), and in the postdrug period (gray). The sampling rate was 24.4 kHz, so that 10 samples correspond to 0.4 ms.

We can maintain memories of stimuli in any sensory modality, including visual, tactile (Romo et al., 1999), and auditory (Rämä et al., 2004) stimuli. Many previous studies focused on the maintenance of visual information. They revealed neuronal correlates for the memorization of multiple visual features, including motion (Mante et al., 2013; Mendoza-Halliday et al., 2014), color (Mohr et al., 2006; Mante et al., 2013), shape (Wilson et al., 1993; Miller et al., 1996; Meyer et al., 2011), and stimulus location (Fuster and Alexander, 1971; Funahashi et al., 1989; Courtney et al., 1997; Fletcher and Henson, 2001; Riley and Constantinidis, 2015). A common task used to probe spatial working memory is the oculomotor delayed-response (ODR) task (Fig. 1*A*), in which subjects keep a location in working memory to make a saccade to it at the end of the trial. Several studies (Fuster and Alexander, 1971; Niki, 1974; Watanabe, 1981; Funahashi et al., 1989; Miller et al., 1996) found that the firing of so-called “delay cells” in the dlPFC of the macaque monkey represents a spatially specific memory trace. They are activated by a visual cue in their receptive field (RF) and remain active during memory delays when the visual cue is extinguished. Delay cells are intermingled with visual cells, which are activated by a visual stimulus but return to baseline when the stimulus is no longer visible. Furthermore, dlPFC lesions cause impairments in working memory (Mishkin, 1957; Grueninger and Pribram, 1969; Bartus and Levere, 1977; Mishkin and Manning, 1978).

The mechanisms underlying persistent activity are only partially understood and may rely on specific circuit and cellular properties. First, persistent firing could involve reverberatory excitation between neurons within (Goldman-Rakic, 1995; Barak and Tsodyks, 2007) or between cortical areas (Fuster et al., 1985; Miller et al., 1996; Chafee and Goldman-Rakic, 2000; Gazzaley and Nobre, 2012; Li et al., 2016). Recent studies revealed an important role for reverberatory interactions between cortex and subcortical structures, including the thalamus and cerebellum (Guo et al., 2017; Gao et al., 2018). Second, persistent firing may at the same time rely on specific membrane conductances that enable sustained excitation of individual neurons induced, for example, by the activation of acetylcholine receptors (Krnjevic et al., 1971; Andrade, 1991; Egorov et al., 2002), dopamine receptors (Vijayraghavan et al., 2007), and noradrenaline receptors (Li et al., 1999; M. Wang et al., 2007). Several studies have also implicated NMDARs in working memory (Lisman et al., 1998; X. J. Wang, 2001). These receptors have a long time constant, and modeling studies suggested that these long time constants are important for stable persistent activity (Lisman et al., 1998; Brunel and Wang, 2001).

Two events need to occur before NMDARs pass current. Glutamate needs to bind, but the neuron also has to be depolarized to release magnesium from the NMDAR channel, which blocks the channel at resting membrane potentials (Hestrin et al., 1990; Daw et al., 1993). In the visual cortex, this gating of NMDARs by membrane depolarization causes them to influence neuronal firing rates multiplicatively, with strong effects on neurons that are well driven by a stimulus and smaller effects for weakly activated cells (Fox et al., 1990; Self et al., 2012). In contrast, AMPARs always depolarize the postsynaptic neurons in an additive manner. It is likely that the gating of NMDA channels also has consequences for persistent activity in higher brain regions, such as frontal cortex. Sensory input to the neurons might release the magnesium block by activating AMPARs so that currents can also flow through NMDA channels, keeping the neurons sufficiently depolarized and thereby causing persistent activity when the stimulus has disappeared. In an elegant study, M. Wang et al. (2013) tested the role of glutamate receptors in persistent firing in the macaque dlPFC. They found that NMDAR antagonists almost abolished persistent activity, whereas the effect of AMPAR antagonists was weak during the start of a delay epoch and only became stronger toward the end of the delay, in support of the specific role of NMDARs in working memory. However, NMDAR blockers had stronger effects than AMPAR blockers in all epochs, making it difficult to rule out that the effects were caused by differences in efficacy of the AMPAR and NMDAR blockers.

In the present study, we tested the hypothesis that AMPARs activate frontal neurons during a sensory stimulus, whereas NMDARs maintain the information as a pattern of persistent firing during a working memory delay. We used microiontophoresis with low ejection currents to perturb neuronal activity without abolishing it, so that we could directly compare the contributions of these receptors in different epochs of the task. We report that AMPARs and NMDARs make comparable contributions to sensory activation and persistent activity. However, the contribution of NMDARs is strongest for the preferred stimulus of a cell, in accordance with their multiplicative effect on neuronal firing rates.

## Materials and Methods

### 

#### 

##### Surgical procedures.

All procedures complied with the National Institutes of Health's *Guide for the care and use of laboratory animals* (National Institutes of Health) and were approved by the institutional animal care and use committee of the Royal Netherlands Academy of Arts and Sciences.

We recorded neural activity from the dlPFC (frontal eye fields and surrounding cortex on the convexity) of 3 adult male macaque monkeys (*Macaca mulatta*: Monkeys B, J, and E). During surgeries, general anesthesia was induced with ketamine (15 mg/kg i.m.) and maintained after intubation by ventilation with a mixture of 70% N_2_O and 30% O_2_, supplemented with 0.8% isoflurane, fentanyl (0.005 mg/kg intravenously), and midazolam (0.5 mg/kg/h intravenously). In a first surgery, the monkeys were implanted with a headpost for head stabilization. The monkeys were then trained on the ODR task until they could reliably perform the task. In a second surgery, we performed a craniotomy (centered on stereotaxic coordinates: 21 mm anterior, and 17 mm lateral) and implanted a titanium chamber (Crist Instruments) for electrophysiological recordings and the iontophoretic administration of the NMDAR antagonist APV and the AMPAR antagonist CNQX. After implantation, the locations of the arcuate and principal sulci relative to the recording chamber were determined using ultrasound imaging (Fig. 1*C*), and the frontal eye fields were localized with electrical microstimulation.

##### Behavioral task.

Monkeys B, J, and E were first trained on the ODR task (Funahashi et al., 1989) (Fig. 1*A*). A fixation point (a red circle of 0.3° diameter) was presented on a gray background, and the monkey started the trial by directing gaze to a 1.5° diameter fixation window centered on the fixation point. After 300 ms of fixation, a visual cue (white circle of 2° diameter) was presented at either the neurons' RF or the antipreferred location (mirrored location relative to the fixation point). After 150 ms, the visual cue was extinguished, but the monkey had to maintain fixation for another 1000 ms before the fixation point was extinguished, which indicated to the monkey that he was required to make a memory-guided eye movement into a target window (4 degrees diameter) that was centered on the location of the previous visual cue. Correct responses were rewarded with apple juice. Trials in which the animal broke fixation before the fixation point was extinguished were aborted, and stimulus conditions were presented in a pseudorandom order. All stimuli were generated using in-house software (Tracker) and presented on a CRT monitor with a resolution of 1024 × 768 pixels and refresh rate of 85 Hz, which was viewed from a distance of 40 cm. Eye movements were recorded with a video eye-tracker (Thomas Recordings) with a sampling rate of 350 Hz.

##### Electrophysiology and iontophoresis.

We recorded single units with tungsten-in-glass electrodes fused with two side barrels (Thiele et al., 2006) (Fig. 1*B*) that were used for iontophoretic drug administration by applying a small electric current to a tungsten wire that was inserted into these side barrels. The impedances of the measuring electrodes ranged from 400 kOhm to 2 MOhm (median ∼1 MOhm) and the impedance of the ejection barrels from 15 to 150 MOhm (median ∼20 MOhm).

The signal from the recording electrode was recorded with Tucker Davis Technology equipment using a high-impedance headstage (RA16AC) and a preamplifier (RA16SD) with a hardware high-pass filter of 2.2 Hz, a low-pass filter of 7.5 kHz (−3 dB point), and sampled with a rate of 24.4 kHz. Spikes were initially determined by setting a voltage threshold. If necessary, spike sorting was done offline using Wave_clus software (Quiroga et al., 2004).

For iontophoresis, we dissolved APV (Sigma Millipore) or CNQX (Sigma Millipore) at 0.02 m in triple-distilled water (pH ∼8.0). APV and CNQX are negatively charged, and we retained them in the glass pipettes by delivering a positive potential (15 nA for APV and 20 nA for CNQX) and ejected them by delivering a negative potential. The ejection currents were set to the amount needed for a noticeable difference in the spiking activity recorded while the monkey performed the ODR task. We adjusted the current to perturb but not abolish the activity, based on the spiking activity heard through a loudspeaker during the experiments. For APV, ejection currents ranged from −2 nA to −7 nA for Monkey B and from −5 nA to −15 nA for Monkey J. For CNQX, ejection currents ranged from −10 nA to −20 nA in both monkeys (J and E). Previous studies demonstrated that iontophoresis of vehicle only has no effect on neuronal firing (Vijayraghavan et al., 2007).

##### RF mapping.

RFs were measured using the same ODR task that was used during the recordings. First, the preferred and antipreferred direction was determined using 8 locations at 8° eccentricity. The eccentricity tuning was subsequently mapped in 4° steps for the preferred and antipreferred direction only. Most of the RFs of the recorded single units were at 18° eccentricity for Monkey B, at 13° eccentricity for Monkey J, and at 18° eccentricity for Monkey E.

##### Data acquisition.

We determined the location of the arcuate sulcus with ultrasound imaging and recorded single-unit activity anterior to this sulcus (Fig. 1*C*). A blunt guide tube, made to tightly fit around the probe, was rigidly attached to a microdrive for mechanical stability (Narishige Scientific). We predimpled the dura with the guide tube and electrode (∼1 mm), penetrated the dura with the electrode, and pulled back the guide tube and electrode to undimple the dura. The electrode was left to settle for ∼20 min. The probe was then carefully advanced until a single unit was encountered. After stabilizing the recording of the spiking activity of the single unit, we determined its RF properties with the ODR task. We only selected isolated single units with spatial selectivity for further recording, and most of these neurons (45 of 57 for the APV dataset, 32 of 51 for the CNQX dataset) showed sustained firing during the memory period. For most single units (47 of 57 for the two APV datasets, 29 of 48 for the two CNQX datasets), three blocks of ∼80 trials were recorded: a recording block of ∼80 trials without drug delivery by maintaining the holding current (from now on called “predrug recordings”), a recording block of ∼80 trials where the drugs was administered by applying the ejection current (“during drug recordings”), and finally a recording block of ∼80 trials without drug delivery, again by maintaining the holding current (“postdrug recordings”). Drug recordings were started once an effect of the drug was noticeable in the spiking activity (monitored through the loudspeaker), usually 3–4 min after the ejection current was applied and the drugs were applied continuously throughout the recording period. Postdrug recordings were started once the effect of drug delivery faded, usually 5–10 min after the holding current was applied after drug delivery. In case of little recovery, the postdrug block was started ∼10 min after cessation of drug application. The waveforms of the recorded spiking activity during one example recording are shown in Figure 1*D*. For a small fraction of the recordings (10 of 57 for the two APV datasets, 13 of 48 for the two CNQX datasets), we lost the single unit during the waiting period after drug delivery so that we could not perform the postdrug recording.

##### Data analyses.

All spike data were binned in bins of 10 ms. The ODR task was divided into two epochs: spontaneous activity and task-related activity. The spontaneous epoch lasted from 300 ms before stimulus onset up to stimulus onset, and the task-related epoch lasted from stimulus onset up to saccade onset. We also evaluated the cue-driven activity in a time window from 50 to 250 ms, persistent activity in a time window from 300 to 1150 ms after cue onset (starting 150 ms after cue offset), and saccade-related activity in a window from 200 ms before the onset of the saccade. To quantify the spatial selectivity for each cell individually, we calculated *d′* for task-related activity as follows:

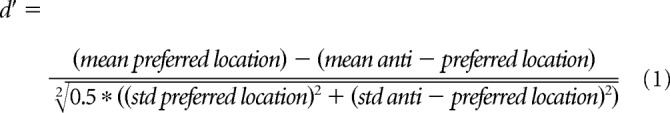
 where std is the SD of the firing rate across trials. For statistical analysis, we used two-sided *t* tests to compare spontaneous and task-related spiking activity between predrug, during drug, and postdrug recordings. A three-way repeated-measures ANOVA with the factors drug (2 levels), epoch (4 levels), and monkey (2 levels) was used to compare drug effects across time windows (based on the average firing rate of individual neurons). Results were considered significant if *p* values were < 0.05 for both monkeys individually as well as when averaged across monkeys.

We investigated whether the influence of APV and CNQX on delay activity in the preferred direction predicted the influence on delay activity in the antipreferred direction by computing the Pearson correlation coefficient *r*. We determined the significance of the difference between correlation coefficients for APV and CNQX by first performing Fischer's *r* to *z* transform and then computing the *z* value of the difference according to the following equation: *z*_Difference_ = (*z*_CNQX_ − *z*_APV_)/sqrt(1/(N_CNQX_ − 3) + 1/(N_APV_ − 3)). The Fano factor was calculated as the variance divided by the mean spiking activity averaged within in a specified time window.

We performed a stratification analysis to control for possibility that a difference in correlation coefficients between APV and CNQX was caused by the relatively low firing rates of cells tested with APV in the antipreferred direction (see Fig. 3*C*,*D*, dashed lines). Such a lower firing rate could have prevented APV from further decreasing activity, and this floor effect could weaken the correlation. We equated the firing rates of cells tested with APV and CNQX in the antipreferred direction by only including a selection of cells in the analysis. We binned cells based on their firing rate in the antipreferred direction (in bins of 5 Hz) and then equated the number of cells tested with APV and CNQX per bin, randomly removing excess cells for one of the drugs (stratification). We recomputed the correlation coefficients for the stratified populations.

## Results

### Behavioral effects of blocking glutamate receptors

**Figure 2.**
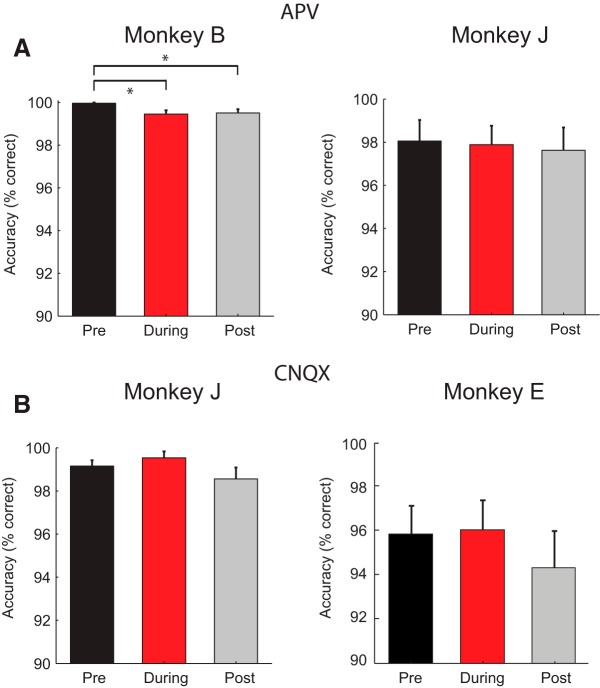
Influence of APV and CNQX on the monkeys' accuracy. ***A***, Accuracy in the ODR task, in the block of trials before APV delivery (black bar), during APV delivery (red bar), and in the postdrug period (gray bar), for Monkey B (left) and Monkey J (right). *p < 0.05. ***B***, Accuracy before CNQX delivery (black bar), during CNQX delivery (red bar), and in the postdrug period (gray bar), for Monkey J (left) and Monkey E (right). Accuracy was relatively high in all conditions. The *y* axis starts at a value of 90%.

At the time we started collecting the data, performance for both monkeys in the ODR task was high (99.9% for Monkey B, 98% during APV recordings and 99.2% during CNQX recordings in Monkey J and 96% for Monkey E) (Fig. 2*A*). To elucidate the contribution of the glutamate receptors to persistent activity during memory delays, we iontophoretically administered the NMDAR antagonist APV or the AMPAR antagonist CNQX. However, we only applied small dosages to perturb activity without abolishing it. At these dosages, the glutamate receptor antagonists did not have consistent effects on accuracy. Although APV decreased the accuracy of Monkey B to 99.4% (*t*_(32)_ = 2.7, *p* = 0.01, paired *t* test), accuracy only slightly increased to 99.5% during the postdrug block (not significantly different from the APV block; *t*_(26)_ = 0.46, *p* = 0.6) (Fig. 2*A*, left), and we cannot exclude the possibility that this decrease in accuracy was caused by a small but systematic decrease in the animal's motivation over time. APV did not influence the accuracy of Monkey J. It was 98% in the predrug epoch, 97.9% during APV administration, and 97.6% in the postdrug block (all *p* values > 0.5) (Fig. 2*A*, right). Similarly, the AMPAR antagonist CNQX application did not influence accuracy in Monkeys J and E (all *p* values > 0.3) (Fig. 2*B*).

### Effects of blocking NMDARs on neuronal activity in the dlPFC

**Figure 3.**
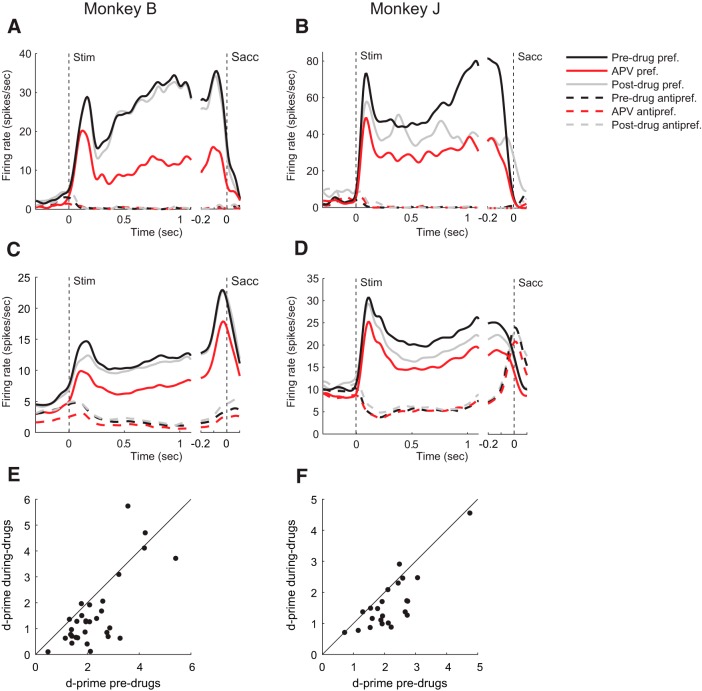
Effect of APV on neuronal activity during ODR task. ***A***, ***B***, Example single units of Monkey B (***A***) and Monkey J (***B***), illustrating the effect of APV neuronal activity. Black trace represents activity in the predrug period. Red trace represents activity after application of APV. Gray trace represents activity in the postdrug epoch. Continuous (dashed) curves indicate activity in the preferred (antipreferred) direction. ***C***, ***D***, The effect of APV on population response of the ODR and recovery for Monkey B (*N* = 33) and Monkey J (*N* = 23). Activity for the preferred location (continuous lines) and antipreferred location (dashed lines), before (black lines) and during APV delivery (red lines). Gray lines indicate activity in the postdrug epoch. ***E***, ***F***, Abscissa, *d′* before APV delivery; ordinate, *d′* during APV delivery. Every data point represents a well-isolated neuron.

To investigate the role of NMDARs in persistent firing, we recorded the activity of single neurons in the dlPFC during the ODR task. We only selected well-isolated single units that exhibited spatial selectivity for further recording. We recorded activity from a total of 56 neurons (33 and 23 neurons in Monkeys B and J, respectively) that were held long enough to compare activity before drug application with that during APV administration. We lost the isolation of 10 neurons (6 in Monkey B and 4 in J) after drug application before the postdrug block, but we were able to record data for the other 46 neurons data during the postdrug block. Most of the neurons (27 of 33 for Monkey B and 15 of 23 for Monkey J) exhibited persistent firing during the memory period, where persistent firing was defined as a persistence index > 2 as follows:

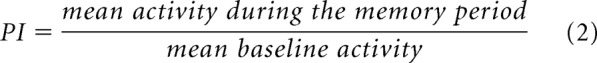
 Typical example recordings for both monkeys are illustrated in Figure 3*A*, *B*, and the population response obtained by averaging across all neurons is shown in Figure 3*C*, *D*. The neurons showed elevated firing during the full duration of the trial when the visual cue was presented at the preferred location of their RF (Fig. 3, continuous curves) while showing a suppression of spiking activity in response to the presentation of the visual cue at the antipreferred location of their RF (dashed curves). In both monkeys, APV suppressed baseline activity before visual cue onset compared with predrug recordings (paired *t* test; Monkey B, *t*_(32)_ = 6.6, *p* = 2 · 10^−7^; Monkey J, *t*_(22)_ = 3.1, *p* = 0.005) (Fig. 3*C*,*D*). Administration of APV suppressed spiking activity in both monkeys at the preferred location during the response elicited by the visual cue (time window 50–250 ms; Monkey B, *t*_(32)_ = 5.1, *p* = 2 · 10^−5^; Monkey J, *t*_(22)_ = 3.0), during persistent activity (time window 300–1150 ms; Monkey B, *t*_(32)_ = 5.2, *p* = 6 · 10^−5^; Monkey J, *t*_(22)_ = 4.7, *p* = 10^−4^) and also in the saccade window (time window 200 ms before saccade; Monkey B, *t*_(32)_ = 3.4, *p* = 0.002; Monkey J, *t*_(22)_ = 2.9, *p* = 0.008) (Fig. 3*C*,*D*). In both monkeys, the suppression was much larger at the preferred location than at the antipreferred location (paired *t* test across neurons in a time window [0, 1150] ms relative to cue onset; Monkey B, *t*_(32)_ = 4.2, *p* = 2 · 10^−4^; Monkey J, *t*_(22)_ = 4.6, *p* = 10^−4^); and in Monkey J, the suppression for cue presentation at the antipreferred location was even absent (one-sample *t* test, *t*_(22)_ = 0.1, *p* = 0.9). Blocking the NMDARs therefore weakened the spatial selectivity of the cells by reducing the difference in spiking activity between the preferred and antipreferred cue. To measure the spatial selectivity, we computed *d′*, which measures how well a single neuron distinguishes between the memory for the two locations in single trials (Eq. 1). APV decreased the *d′* for most cells in both monkeys (Fig. 3*E*,*F*) during the memory epoch (300–1150 ms after the onset of the cue, i.e., starting 150 ms after cue offset). In Monkey B, the *d′* decreased from an average of 2.42 to a value of 1.69 (*t*_(32)_ = 4.7, *p* = 5 · 10^−5^, paired *t* test) and in Monkey J, *d′* decreased from 2.17 to 1.64 (*t*_(22)_ = 5.0, *p* = 5 · 10^−5^).

Spiking activity gradually restored to predrug levels when APV administration was stopped. Although recovery was not complete in all our recordings, the activity of all the cells changed back into the direction of predrug recordings, both for baseline spiking activity before visual cue onset (paired *t* test; Monkey B, *t*_(26)_ = 2.5, *p* = 10^−3^; Monkey J, *t*_(18)_ = 2.7, *p* = 0.02) as well as for spiking activity for the remainder of the trial when the visual cue was presented at the preferred (50–1150 ms after cue-onset; Monkey B, *t*_(26)_ = 3.4, *p* = 0.002; Monkey J, *t*_(18)_ = 1.1, *p* = 0.3) and antipreferred location for Monkey B (*t*_(26)_ = 3.2, *p* = 0.003) (Fig. 3*C*). In Monkey J, the suppression of spiking activity following APV delivery was absent for the antipreferred direction, and we also did not observe a restoration of spiking activity for this direction (*t*_(18)_ = 1.2, *p* = 0.2) (Fig. 3*D*). To investigate whether a change in the variability of the neuronal response across trials contributed to this decrease in the *d′*, we also computed the influence of APV on the Fano factor (time window 300–1150 ms). In Monkey B, APV did not have a significant effect on the Fano factor during the delay period, neither for the preferred direction (*t*_(32)_ = 2.0, *p* = 0.052, paired *t* test) nor for the antipreferred direction (*t*_(32)_ = 1.5, *p* = 0.2). In Monkey J, APV increased the Fano factor for the preferred direction (*t*_(22)_ = 2.9, *p* = 0.009), but not for the antipreferred direction (*t*_(22)_ = 0.24, *p* = 0.8).

**Figure 4.**
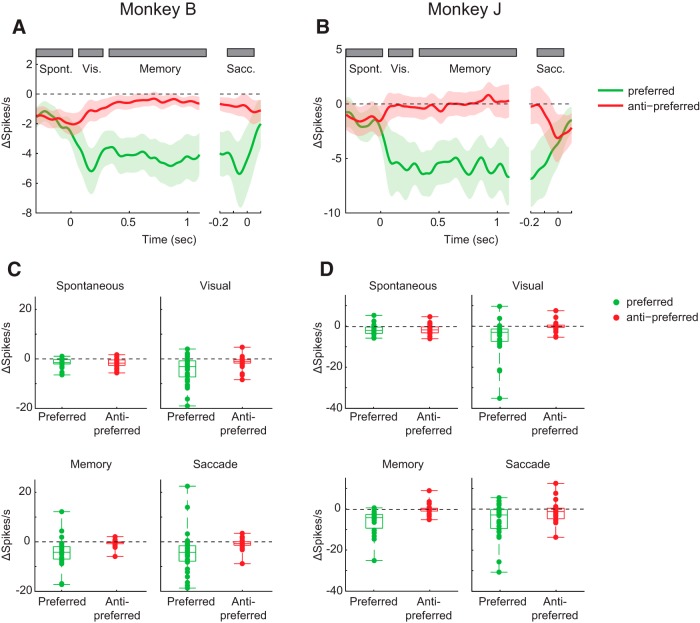
Effect of APV on neuronal activity for the preferred and nonpreferred direction. ***A***, ***B***, APV-induced difference in spiking activity during the ODR task, for Monkey B (***A***) and Monkey J (***B***). We subtracted the activity in the predrug period from that during APV delivery, elicited in trials with a cue at the preferred (green trace) and antipreferred location (red trace). Gray rectangles represent the time windows used for quantification. ***C***, ***D***, Effect of drugs on individual single units in the four trial epochs for Monkey B (***C***) and Monkey J (***D***).

**Figure 5.**
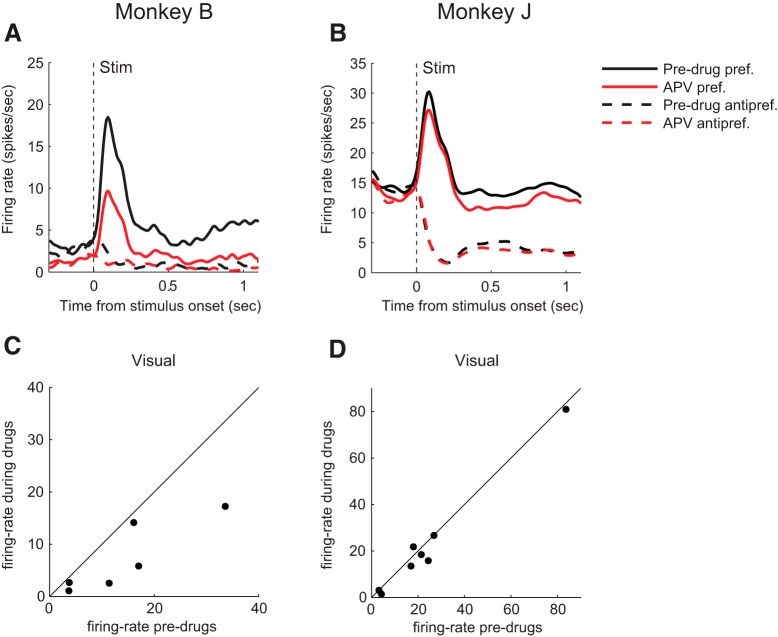
Effect of APV on the activity of visual cells. ***A***, ***B***, Average response of visual neurons without delay activity in Monkey B (*N* = 6) (***A***) and Monkey J (*N* = 8) (***B***), elicited in trials with a cue at the preferred (continuous) and antipreferred location (dashed), before (black traces) and during APV delivery (red traces). ***C***, ***D***, Abscissa, activity elicited by the preferred cue before APV delivery; ordinate, activity during APV delivery. Every data point represents an individual visual cell.

To further examine the time course of the drug effect, we plotted the difference between spiking activity before and during the administration of APV (Fig. 4*A*,*B*). For the preferred location, we compared the effects of APV during different epochs of the task (spontaneous, visual, delay, and saccade activity) for the 2 monkeys using a three-way repeated-measures ANOVAs with the factors drug (2 levels), epoch (4 levels), and monkey (2 levels) (Fig. 4*C*,*D*). As expected, we observed a main effect of APV on the firing rate (*F*_(3,435)_ = 9.51, *p* = 0.0022). However, there was no significant interaction between the drug effect and the epoch, indicating that there was no difference in the drug effect between epochs. The decrease of the cue-driven response is in accordance with a general multiplicative effect of NMDARs on spiking activity but appears to be at odds with the hypothesis that NMDARs have a specific role in the generation of persistent activity. We therefore also examined the small subset of neurons with a visual response without delay activity (*N* = 6 in Monkey B and *N* = 8 in Monkey J) (Fig. 5*A*,*B*). APV suppressed the visually driven activity of these neurons in both monkeys (paired *t* test; Monkey B, *t*_(5)_ = 3.0, *p* = 0.03; Monkey J, *t*_(7)_ = 2.5, *p* = 0.04), in accordance with a more general role of NMDARs in both cue-driven and persistent activity (Fig. 5*C*,*D*).

### Contribution of AMPARs to activity in dlPFC

We recorded a total of 41 neurons during CNQX application (27 in Monkey J and 14 in Monkey E), and more than half of them exhibited sustained firing during the memory period (15 of 27 for Monkey J and 14 of 14 for Monkey E). Of these neurons, 29 were kept long enough to examine activity in the postdrug period.

**Figure 6.**
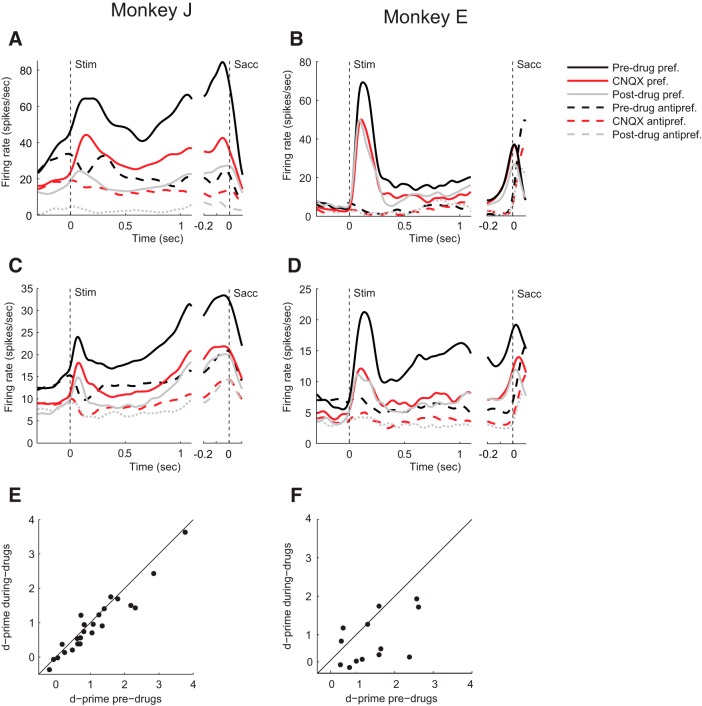
Effect of CNQX on neuronal activity. ***A***, ***B***, The influence of CNQX on neuronal activity in example neurons in Monkey J (***A***) and Monkey E (***B***). Black curves indicate the predrug period. Red curves indicate activity after CNQX application. Continuous curves indicate the preferred cue. Dashed curves indicate the nonpreferred cue. Gray curves indicate the postdrug epoch. ***C***, ***D***, Average activity of neurons in Monkey J (*N* = 27) (***C***) and Monkey E (*N* = 14) (***D***). ***E***, ***F***, Abscissa, *d′* before CNQX delivery; ordinate, *d′* during CNQX delivery.

Typical example recordings are illustrated in Figure 6*A*, *B*, and the population response is shown in Figure 6*C*, *D*. In both monkeys, baseline spiking activity before visual cue onset was suppressed during the CNQX administration (paired *t* test; Monkey J, *t*_(26)_ = 4.6, *p* = 10^−4^; Monkey E, *t*_(13)_ = 2.3, *p* = 0.04) (Fig. 6*C*,*D*). For both monkeys, administration of CNQX suppressed spiking activity in the cue window (paired *t* test; Monkey J, *t*_(26)_ = 3.8, *p* = 9 · 10^−4^; Monkey E, *t*_(13)_ = 4.1, *p* = 0.001) and memory window (Monkey J, *t*_(26)_ = 4.4, *p* = 2 · 10^−4^; Monkey E, *t*_(13)_ = 3.5, *p* = 0.004) when the visual cue was presented at the preferred location. A similar effect was observed in the saccade window (Monkey J, *t*_(26)_ = 4.7, *p* = 7 · 10^−5^; Monkey E, *t*_(13)_ = 2.4, *p* = 0.03). When the visual cue was presented at the antipreferred location, there was also a significant reduction of activity in the cue window (Monkey J, *t*_(26)_ = 3.7, *p* = 0.001; Monkey E, *t*_(13)_ = 3.1, *p* = 0.009). The reduction of activity in the antipreferred direction was significant in Monkey J in the memory window (*t*_(26)_ = 3.3, *p* = 0.003) and in the saccade window (*t*_(26)_ = 3.2, *p* = 0.003), but not in Monkey E (memory/saccade window, both *p* values > 0.15) (Fig. 6*C*,*D*). To examine the influence of CNQX on the spatial selectivity, we calculated *d′* values. In Monkey J, CNQX caused a decrease in *d′* from an average of 1.01 to a value of 0.83 (paired *t* test, *t*_(26)_ = 3.3, *p* = 0.003); and in Monkey E, the *d′* decreased from 1.32 to 0.82 (*t*_(13)_ = 2.9, *p* = 0.01) (Fig. 6*E*,*F*). We next investigated the influence of CNQX on the Fano factor during the delay period. In both monkeys, CNQX did not have a significant effect on the Fano factor, neither in the preferred direction (paired *t* test; Monkey J, *t*_(26)_ = −0.1, *p* = 0.9; Monkey E, *t*_(13)_ = 0.8, *p* = 0.4) nor in the antipreferred direction (Monkey J, *t*_(26)_ = −0.06, *p* = 0.95; Monkey E, *t*_(13)_ = 2.0, *p* = 0.06).

Spiking activity did not restore to predrug levels when CNQX administration ceased (Fig. 6*A–D*, gray curves). Some single units (9 of 19 for Monkey J, 4 of 14 for Monkey E) did not even show a trend of recovery; and at the population level, recovery was not evident either. The absence of recovery is in accordance with previous studies showing that CNQX has long-lasting effects (Leininger and Belousov, 2009; Self et al., 2012).

**Figure 7.**
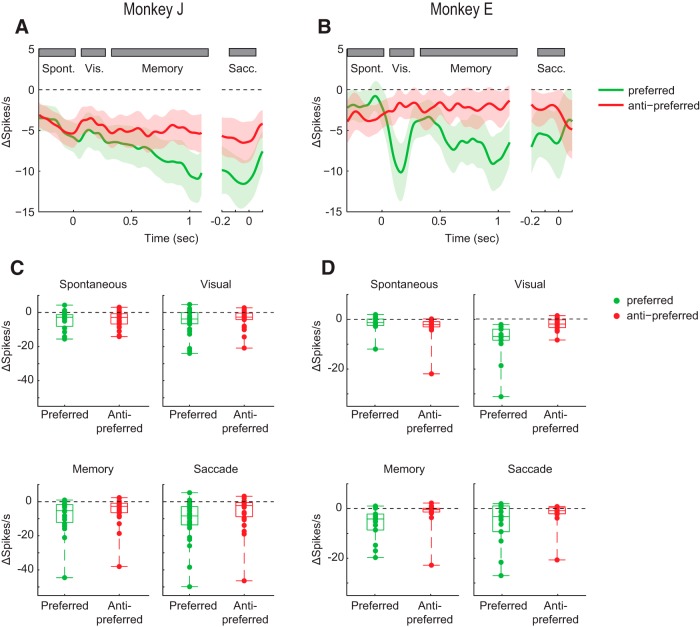
Effect of CNQX on neuronal activity elicited by cues at the preferred and nonpreferred location. The influence of CNQX on the neuronal responses was determined by subtracting neuronal activity in the predrug period from that elicited when CNQX was applied, in Monkey J (***A***) and Monkey E (***B***). Green trace represents activity difference elicited by the preferred cue. Red trace represents activity elicited by the antipreferred cue. ***C***, ***D***, Boxplots of the decrease in activity caused by CNQX in the four epochs for Monkey J (***C***) and Monkey E (***D***).

To examine the time course of the AMPAR contribution, we determined the difference between spiking activity before and during the administration of CNQX (Fig. 7). A three-way ANOVA with factors epoch, drug/no-drug, and monkey for the preferred cue location revealed that the influence of CNQX on the firing rate was significant (*F*_(3,315)_ = 17.8, *p* < 0.001), but that there was no interaction effect between drug and epoch (*F*_(3,315)_, *p* = 0.76), indicating that the drug effect was similar across epochs.

### Comparison of the effect of APV and CNQX on delay activity

**Figure 8.**
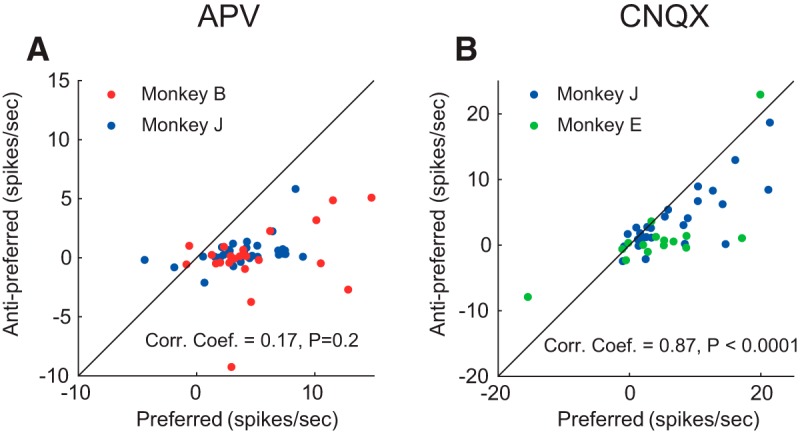
Comparison of effects on delay activity at the preferred and nonpreferred location for APV and CNQX. Reduction in firing rate (predrug − drug) during the delay period for the preferred cue (*x* axis) and antipreferred cue (*y* axis) during the application of APV (***A***) and CNQX (***B***).

A comparison of the effects of APV and CNQX revealed that the reduction in *d′* was larger for NMDAR than for AMPAR administration (*t*_(94)_ = 2.6, *p* = 0.01, two-sample *t* test). However, we cannot draw strong conclusions from this comparison because there were differences between ejection currents, and effects of APV and CNQX also depend on a number of poorly controlled factors, including the efficiency of the drug, the distance between the neuron and pipette, and the diffusion and clearance of the drugs. A better approach is to determine how well the influence on delay activity for the preferred cue predicts the influence on delay activity for the nonpreferred cue, by computing the correlation (Fig. 8).

The correlation coefficient for APV was 0.17, which was not significant (*N* = 56 cells with sufficient delay activity; *p* = 0.2). Thus, the activity decrease in the preferred direction was a relatively poor predictor of the activity decrease in the antipreferred direction. The correlation coefficient for CNQX was 0.87, which was significant (*N* = 41 cells; *p* < 0.0001), indicating that the prediction worked much better for CNQX. Indeed, the difference between the magnitude of correlation coefficients for APV and CNQX was also significant (*z* = 5.5, *p* = 4 · 10^−8^; Fischer's *r* to *z* transform; see Materials and Methods). We considered the possibility that the weak correlation for APV could have been caused by the relatively low firing rates of cells tested with this drug in the antipreferred direction (Fig. 3*C*,*D*, dashed lines), preventing APV from further decreasing activity (a floor effect), and weakening the correlation. We therefore performed a stratification analysis that equates firing rates in the antipreferred direction of cells tested with APV and CNQX (described in Materials and Methods). After stratification, 29 cells tested with APV and 29 cells tested with CNQX remained. In the population with equated firing rates, the correlation coefficient for CNQX of 0.63 was higher than the value of 0.06 for APV (*p* = 0.01). We conclude that the difference in the correlation coefficients between drugs was not caused by a floor effect.

Instead, it seems likely that this difference between APV and CNQX is caused by the distinct actions of AMPARs and NDMARs. When glutamate binds to an AMPAR, the channel opens and the cell is activated. In contract, magnesium blocks NMDARs when the cell is not sufficiently depolarized. This magnesium block may explain why a decrease in delay activity in the preferred direction was not always accompanied by a comparable decrease in the antipreferred direction. The NMDAR may have still been blocked by magnesium in the antipreferred direction due to the lower neuronal firing rate, so that APV could not exert its effect.

## Discussion

In this study, we investigated the contribution of AMPARs and NMDARs to visually evoked activity and persistent firing in dlPFC. We iontophoretically applied antagonists of AMPARs and NMDARs using relatively small ejection currents to perturb activity without entirely blocking it to obtain sensitive measures of the role of the receptors during different epochs of a delayed saccade task. Although the blockade of glutamate receptors had substantial effects on spiking activity, we did not find consistent effects on the monkeys' accuracy (Fig. 2), similar to many previous studies using iontophoretic drug application (Vijayraghavan et al., 2007; M. Wang et al., 2013). This absence of an effect on accuracy is expected as iontophoretically applied drugs do not spread far (Kelly and Renaud, 1974; Rao et al., 2000), so that we caused a relatively weak perturbation in the activity of a small population of neurons.

We found that AMPARs and NMDARs contribute to neuronal activity during all phases of the ODR task. The similarity of the effects AMPA and NMDA blockers on visually evoked activity and persistent activity differs from a previous study examining texture segregation in area V1, where AMPA blockers mainly decreased the visually driven activity, whereas NMDA interfered specifically with the enhanced representation of figural texture elements over the background (Self et al., 2012), which is caused by feedback from higher cortical areas (Klink et al., 2017). In the present working memory task, the effects of AMPARs were largely additive because the decrease in spiking activity caused by CNQX was substantial in the preferred as well as in the antipreferred direction (Fig. 6*C*,*D*). Furthermore, the reduction of activity elicited by the cue in the neurons' preferred direction predicted the decrease in activity in the antipreferred direction relatively well (Fig. 8). The effects of blocking AMPARs were prominent in the baseline epoch, during the cue period, the memory delay, and also around the time of the saccade. In contrast, NMDARs contributed strongly to the activity of well-driven neurons and less to the firing rate of weakly activated cells (Fig. 3*C*,*D*). Accordingly, the decrease of activity elicited by the preferred cue caused by APV was a poor predictor for the decrease in activity for the nonpreferred direction (Fig. 8). Our finding that NMDARs amplify the activity of well-driven neurons whereas the influence of AMPARs tend to be additive is compatible with previous results in the cat visual cortex (Fox et al., 1990; Sato et al., 1999).

These differential effects of AMPARs and NMDARs also explained the difference of the effects of APV an CNQX on the reliability of the spatial selectivity. Blocking AMPARs caused a relative moderate decrease of the *d′* because it decreased activity elicited by cues at the preferred and antipreferred locations similarly so that the *d′* decreased only slightly (Fig. 6*E*,*F*). In contrast, blocking NMDARs strongly reduced the *d′* because the decrease in activity elicited by the neurons' preferred cue was more pronounced than that elicited by the nonpreferred cue (Fig. 3*E*,*F*).

A previous study by M. Wang et al. (2013) suggested that persistent activity relies on the unique properties of the NMDAR, with its voltage-dependent gating due to the magnesium block and its relatively long time constant. They observed that NMDARs enable persistent activity during the entire delay period of a working memory task, whereas AMPARs had little effect on persistent activity during the early delay period and a stronger effect later during the delay. The idea is that the unique properties of NMDARs cause a positive feedback loop between membrane depolarization and the release of the magnesium block, so that the excitatory currents can outlast a transient input onto the cell (M. Wang et al., 2013). In accordance with that study, we also found that NMDARs had a strong effect on persistent activity and that the contribution of NMDARs was particularly strong for the preferred direction. However, our results do not support a specific role of NMDARs in persistent firing, for a number of reasons. First, we found that AMPARs also strongly contributed to persistent activity throughout the memory epoch (Fig. 6*C*,*D*). Second, the contribution of NMDARs to the initial visual response and to saccade-related activity was comparable with the contribution to the delay activity (Fig. 3*C*,*D*). Third, blocking of NMDARs also reduced spiking activity and weakened the spatial selectivity of visual cells without persistent activity (Fig. 5).

At first sight, our results are therefore at odds with the results of M. Wang et al. (2013). One difference between studies was in the choice of antagonists. We used the competitive NMDAR antagonist APV, whereas M. Wang et al. (2013) used MK801, which is a noncompetitive antagonist, and the NMDA subunit antagonist Ro25–6981, which blocks NMDARs with the NR2B subunit. Furthermore, we used CNQX to block AMPRA-Rs, whereas M. Wang et al. (2013) used both NBQX and CNQX. However, we believe that the most important difference between studies is in the dosage of the drugs. M. Wang et al. (2013) almost completely abolished delay activity with the application of NMDA antagonists but observed weaker effects with AMPAR antagonists. It is likely that they would have seen a more complete suppression of delay activity with higher dosages of AMPA blockers because, in our experience, higher dosages of CNQX can also completely block neuronal activity. In the present study, we rather used iontophoresis currents for the NMDA and AMPA antagonists that perturb but do not abolish activity, and we observed that both antagonists had comparable effects on persistent neuronal activity. It is also of interest to compare the present results with a study by Skoblenick and Everling (2012) who recorded from the dlPFC during a systematic dose of the NMDA antagonist ketamine. Ketamine increased, rather than decreased, the activity of most dlPFC neurons, in accordance with studies in the frontal cortex of rodents (Homayoun and Moghaddam, 2007). This discrepancy is most likely related to the systematic application of ketamine, which influences neuronal activity in many brain regions that can indirectly impact on the activity of neurons in the dlPFC. In the present and previous studies (M. Wang et al., 2013), the local, iontophoretic application of NMDA blockers invariably decreased neuronal activity in dlPFC.

AMPARs and NMDARs are not the only receptors that have been implicated in the mechanisms for persistent firing. Blocking dopamine receptor D1, for instance, revealed an “inverted U” dose-response relationship because too little or too much receptor activity reduces persistent firing (Vijayraghavan et al., 2007), and both D1 and D2 receptors influence the representation of task rules during a delay (Ott et al., 2014). Similarly, acetylcholine has been implicated in the maintenance of persistent activity through its action on nicotinic (Yang et al., 2013; Sun et al., 2017) and muscarinic receptors (Krnjevic et al., 1971; Andrade, 1991; Egorov et al., 2002), although a recent study demonstrated that the decrease in activity caused by muscarinic blockers is not specific to delay activity (Major et al., 2015), just as we observed for NMDARs. Furthermore, a2A-adrenoceptors (Li et al., 1999; M. Wang et al., 2007) also impact on persistent firing, in part by acting on nonselective cation-permeable transient receptor potential channels (TRP channels) (Yan et al., 2009) and hyperpolarization activated cyclic nucleotide-gated potassium channels (M. Wang et al., 2007; Thuault et al., 2013). Thus, many receptors contribute to persistent firing, implying a complex interplay between many receptors, including NMDARs and AMPARs.

The activation of the receptors that cause persistent firing requires synaptic input that might be provided by other neurons with persistent activity within the same area (Goldman-Rakic, 1995; Barak and Tsodyks, 2007) and from reciprocal excitatory loops between cortical and/or subcortical areas (Fuster et al., 1985; Miller et al., 1996; Chafee and Goldman-Rakic, 2000; Gazzaley and Nobre, 2012; Guo et al., 2017; Gao et al., 2018). In the first, local scenario, the persistent firing would be generated by reciprocal excitation between pyramidal neurons with similar tuning in the same area. In the second, more global scenario, persistent firing is maintained by reciprocal excitation between cortical areas or by loops through subcortical structures, including the thalamus (Guo et al., 2017; Jaramillo et al., 2019), cerebellum (Gao et al., 2018), and/or basal ganglia (Kawagoe et al., 1998). Persistent activity during memory delays is indeed observed in many other cortical areas (Christophel et al., 2017), including the parietal cortex (Colby et al., 1996; Chafee and Goldman-Rakic, 1998), medial superior temporal cortex (Mendoza-Halliday et al., 2014), the inferotemporal cortex (Fuster and Jervey, 1981, 1982; Miyashita, 1988), and even in the primary visual cortex (Supèr et al., 2001; van Kerkoerle et al., 2017). A study by Chafee and Goldman-Rakic (2000) of monkeys combined local cooling of either parietal and PFC with recording in the other area during a working memory task. The inactivation of one area decrease the activity of some neurons in the other region but increased the activity of others, without a clear effect on behavior. Recent studies that approached the same questions in mice revealed an important role of frontal cortex in the maintenance of information during memory delays (Ren et al., 2014; Goard et al., 2016; Li et al., 2016). Optogenetic silencing of neuronal activity in the frontal cortex during memory delays was able to delete working memories. Interestingly, a brief unilateral blockade of persistent activity in frontal cortex could be later restored by activity of the contralateral frontal cortex, in accordance with the hypothesis that persistent activity relies on the reverberation of activity between areas of the cerebral cortex (Li et al., 2016). Furthermore, the persistent activity of neurons in the frontal cortex could also be abolished by optogenetic inhibition of the thalamus or the cerebellar nuclei; and vice versa, cortical silencing abolished persistent firing in thalamus and the cerebellar nuclei. Hence, these recent results directly demonstrated a crucial role for loops between cortex and these subcortical structures in the maintenance of persistent firing (Guo et al., 2017; Gao et al., 2018).

In combination with these previous studies, the present results contribute to our understanding of how working memories are maintained in the frontal cortex, revealing that both AMPARs and NMDARs sustain persistent spiking activity, although the relative contribution of NMDARs increases for neurons that are strongly active. This is a relevant finding, both for models on the neural mechanisms during working memory (Lisman et al., 1998; Compte et al., 2000; Brunel and Wang, 2001; X. J. Wang, 2001) and for clinical conditions in which working memory is impaired. Future experimental and modeling studies can now investigate how the dynamics of these receptors, in combination with recurrent excitation within and between brain regions, explain how task-relevant information is kept online during memory delays.
